# Structure and Composition of Spermatozoa Fibrous Sheath in Diverse Groups of Metazoa

**DOI:** 10.3390/ijms25147663

**Published:** 2024-07-12

**Authors:** Ekaterina A. Guseva, Vitaly S. Buev, Sabina E. Mirzaeva, Philipp I. Pletnev, Olga A. Dontsova, Petr V. Sergiev

**Affiliations:** 1 Center of Life Sciences, Skolkovo Institute of Science and Technology, 143025 Skolkovo, Russia; eguseva98@mail.ru (E.A.G.); olga.a.dontsova@gmail.com (O.A.D.); 2 Belozersky Institute of Physico-Chemical Biology, Lomonosov Moscow State University, 119991 Moscow, Russia; 3 Faculty of Chemistry, Lomonosov Moscow State University, 119991 Moscow, Russia; vittaria.fern@gmail.com (V.S.B.); sabina.mirzaeva@chemistry.msu.ru (S.E.M.); philippletnev@gmail.com (P.I.P.); 4 Faculty of Bioengeneering and Bioinformatics, Lomonosov Moscow State University, 119991 Moscow, Russia; 5 Shemyakin–Ovchinnikov Institute of Bioorganic Chemistry, 117997 Moscow, Russia

**Keywords:** spermatozoa, fibrous sheath, longitudinal columns, flagella ultrastructure, evolution of fibrous sheath

## Abstract

The proper functioning and assembly of the sperm flagella structures contribute significantly to spermatozoa motility and overall male fertility. However, the fine mechanisms of assembly steps are poorly studied due to the high diversity of cell types, low solubility of the corresponding protein structures, and high tissue and cell specificity. One of the open questions for investigation is the attachment of longitudinal columns to the doublets 3 and 8 of axonemal microtubules through the outer dense fibers. A number of mutations affecting the assembly of flagella in model organisms are known. Additionally, evolutionary genomics data and comparative analysis of flagella morphology are available for a set of non-model species. This review is devoted to the analysis of diverse ultrastructures of sperm flagellum of Metazoa combined with an overview of the evolutionary distribution and function of the mammalian fibrous sheath proteins.

## 1. Introduction

**Figure 1:**
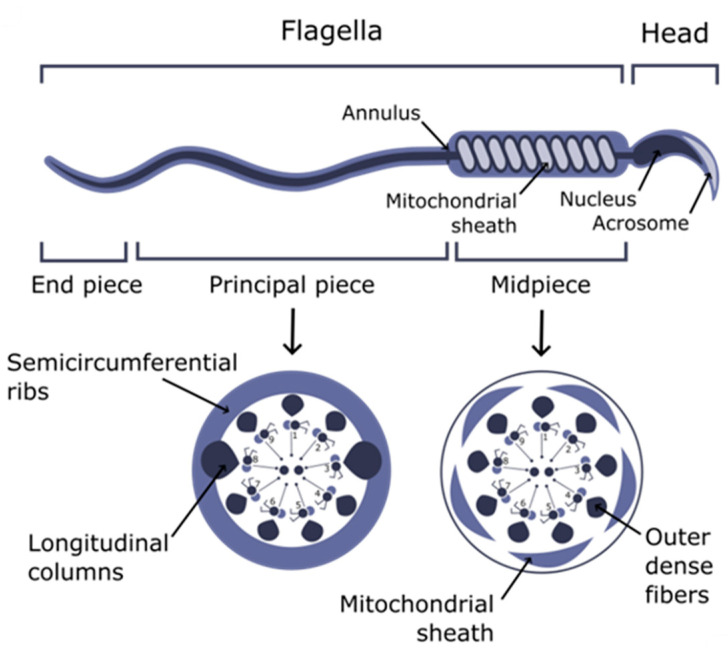
The scheme of mouse spermatozoa morphology and flagella ultrastructures.

Metazoa is a large group that includes from 3 to 30 million multicellular animal species, according to various estimates. Almost every Metazoan phylum contains animal species possessing motile germ cells. The most common example of such cells is spermatozoa. These cells are morphologically distinct from all the other cells of the organism and consist of two main parts: the head carrying genetic material and acrosome; and the tail responsible for the motility [1] (Figure 1). The structure of the tail includes the following parts: connecting pieces located between the head and tail; midpiece containing mitochondrial sheath; principal piece; and short end piece. Although the diversity of spermatozoa in general is quite large, many conservative features may be observed in the structure of their flagella.

A vast majority of animals have a typical structure of the sperm flagellum, which usually includes a classical axoneme originating from the distal centriole. Axoneme consists of microtubules organized in 10 doublets: one is located in the center, and others in a circle on the periphery. Typically, a formula 9 × 2 + 2 is used to describe the axoneme organization [2]. Microtubules in a doublet differ: only microtubule A carries dynein arms and radial spokes on its surface, while microtubule B is associated with A [3]. 

**Figure 2:**
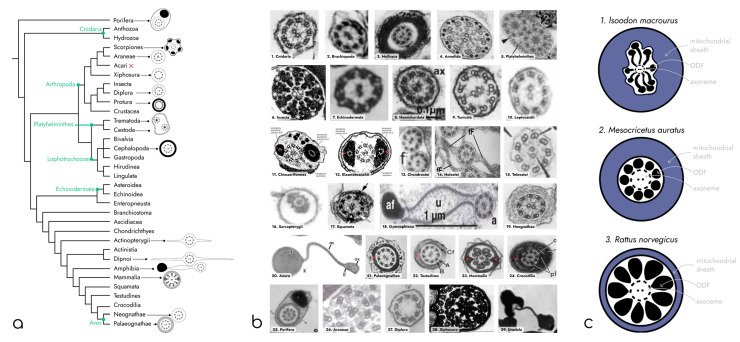
(**a**) Phylogenetic tree [6] of main groups of Metazoa with schematic representation of the spermatozoa principal piece composition in selected groups. (**b**) Diversity of sperm flagella principal piece structure. 1—Cnidaria [7], 2—Brachiopoda [8], 3—Mollusca [9], 4—Annelida [10], 5—Platyhelminthes [11], 6—Insecta [12], 7—Echinodermata [13], 8—Hemichordata [14] (ax—axoneme), 9—Tunicata [14], 10—Leptocardii [15], 11—ord. Chimaeriformes [16], 12—Elasmobranchii [17], 13—Chondrostei [18], 14—Holostei [19] (fF—flagellar fins), 15—Teleostei [20], 16—Sarcopterygii [21], 17—Squamata [22], 18—Gymnophiona [23] (a—axoneme, u—undulating membrane, af—axial fiber), 19—Neognathae [24], 20—Anura [25], 21—Palaeognathae [26], 22—Testudines [27] (Cf—concentric fibers), 23—Mammalia [28], 24—Crocodilia [29], 25—Porifera [30] (ax—axoneme, n—nucleus), 26—Araneae [31], 27—Diplura [32], 28—Xiphosura [33], 29—Urodela [34]. MTB—microtubules. Red arrows indicate FS. (**c**) Schemes of cross-sectional sizes of ODF of 1—*Isoodon macrourus* [35], 2—*Mesocricetus auratus* [36], 3—*Rattus norvegicus* [36].

In different groups of animals, the axoneme may be surrounded by diverse structural elements (Figure 2a). For example, in mammals, outer dense fibers (ODF) and fibrous sheath (FS) are located outside the axoneme. The fibers of ODF start in the midpiece and reach the distal end of the principal piece. At the same time, the fibrous sheath is present exclusively in the principal piece and lies between the plasma membrane and ODF. Usually, circumferential ribs and longitudinal columns are distinguished in the FS structure. Longitudinal columns are attached to the doublets 3 and 8 of microtubules through the ODF and connected together by the semicircumferential ribs [3]. All these structures play an important role in motility, signaling, and even in the sperm energy supply system, as they provide a scaffold for the enzyme anchoring [4,5].

The assembly of tail structural elements is poorly studied. However, it was shown that tubulin modifications, such as polyglutamylation, play a crucial role in this process [37,38]. One of the most intriguing open questions is the mechanism of longitudinal columns positioning to the doublets 3 and 8 of microtubules. An evolutionary-conserved system might be responsible for it, as several homologous structures in other animal species are also localized in the same way. 

## 2. Diversity of the Structure of Sperm Flagella in Metazoa

### 2.1. Unusual Sperm Shapes

In the basal group of the Metazoa, Porifera, spermatozoa are characterized by a unique V-shaped form. This type of morphology results in unusual organelle localization: the flagellum is surrounded by a cytoplasmic tunnel and, up to the middle of its length, is closely adjacent to the nucleus (Figure 2(b25)). For this type of sperm, the rotational model of movement was proposed, in which the sperm head plays the role of a stabilizer of a gravity center [30]. Also, the same V-shaped structure can be observed in group Phoronida [39].

What is even more interesting is that sperm cells may contain two flagella in some groups of animals, for example, Platyhelminthes. The overall shape of such spermatozoa differs from the usual one [11]. The mitochondria appear in the middle part, and the nucleus is localized in the end part of the sperm cell. Two axonemes go throughout the cell and are surrounded by a layer of cortical microtubules on the proximal end [11].

The other example of biflagellate sperm may be found in the class of ray-finned fish, in particular, the subclass Cladistia. It is assumed that the second flagellum arose several times independently in the evolution of fish (Figure 2(b13)) [18].

### 2.2. Diversity of Axoneme Anchoring Structures

An unusual type of axoneme anchoring is widespread among representatives of the class Hirudinea. Their flagella completely lack the distal centriole, which is needed for anchoring the axoneme. However, the central pair of their axonemal microtubules is surrounded by an electron-dense sheath, which is thought to perform the same function as a distal centriole in other animals [40].

In contrast, the flagella of members of phylum Echinodermata are characterized by additional pericentriolar structures, which are associated with their distal centriole (Figure 2(b7)). They have a tubular structure and originate between microtubular triplets of centrioles. Near the plasma membrane, each of these tubular protrusions branches into three parts, each of which further fuses with the plasma membrane [13].

### 2.3. Diversity of Axoneme Organization

A great diversity of axonemal organizations can also be found among representatives of the animal world. For example, biflagellate spermatozoa of *Opisthorchis viverrini* (phylum Platyhelminthes) (Figure 2(b5)) contains two axonemes, each of which possesses only one central microtubule (9 ×2 + 1) [11]. Moreover, some representatives of the order Xiphosura, for example, *Carcinoscorpius rotundicaud*, have completely lost the central pair of microtubules (9 × 2 + 0) [32] (Figure 2(b27)).

In contrast, the other group of animals demonstrates an increase in the number of microtubules in the axoneme. For example, spermatozoa of representatives of the order Diplura and Diptera have axoneme with nine additional auxiliary microtubules (9 + 9 × 2 + 2) [41]. Each additional microtubule of the Diptera consists of 13 protofilaments and is associated with one doublet of the classical axoneme and two electron-dense masses (Figure 2(b6)) [12,42]. In the tail of the Diplura sperm, auxiliary microtubules lie outside of doublets 6–9 (Figure 2(b28)) [33]. An even more fascinating type of axoneme may be seen in Araneae. These animals have a central triplet (9 × 2 + 3) (Figure 2(b26)) instead of a classical doublet [31].

Also, an interesting phenomenon was observed in the ultrastructure of the ocean fish *Macrozoarces americanus*. It was shown that besides the usual organization of the axoneme, a portion of its sperm tails contained an irregular microtubule arrangement with additional doublets [20]. The significance of this type of arrangement remains unknown.

### 2.4. Structures Surrounding Axoneme

The structures surrounding the flagella axoneme are also of interest, as they affect sperm motility. In most invertebrate animals, the axoneme is surrounded only by a plasma membrane, which can be smooth or form lateral projections, as in Actinopterygii [43], phylum Brachiopoda [8], and the Echinodermata [13] (Figure 2(b3,7,14)). 

Additional electron-dense granular structures that contain glycogen are observed outside the axoneme in sperm cells of types Mollusca (Figure 2(b3)) and Annelida (Figure 2(b4)) [9,10,44]. This additional part of the sperm tail is characterized by the association of glycogen granules with a central pair of microtubules or intra-axonemal dense granules [44]. Moreover, in some gastropods, microtubules in the glycogen fragment disappear [9].

The axoneme of the vast majority of representatives of phylum Chordata is surrounded by a fibrous sheath. The fibrous sheath is the electron-dense protein layer of the flagellum that underlies the plasma membrane and surrounds the outer dense fibers and axoneme. It is located in the main part of the flagellum, which makes up about three-quarters of its entire length [45]. The fibrous membrane consists of two longitudinal columns connected by semicircular ribs. Longitudinal columns (LC) are attached to outer dense fibers 3 and 8 in the anterior part of the principal piece, while in the middle and posterior part of the principal piece, they are associated with doublets 3 and 8 of microtubules [3]. The attachment of the LC to doublets 3 and 8 of microtubules appears to limit the participation of these microtubules in the gliding movements that are responsible for bending the flagellum [3]. The longitudinal columns apparently allow for bending in a plane passing through the central pair of microtubules of the axoneme, but they are difficult to bend in a plane perpendicular to it. Narrowing of the longitudinal columns and thinning of the peripheral ribs may reduce their influence on the bending of the flagellum in the distal part of the tail, where it is capable of moving in all three planes [3]. Also, the proteins of the fibrous sheath are involved in signaling, which will be discussed below.

However, in some vertebrates, the fibrous sheath and all associated structures are absent, for example, in the subtype Tunicata (Figure 2(b9)) [46] and the classes Leptocardii (Figure 2(b10)) [15] and Actinopterygii (Figure 2(b13–15)) [18,20,43]. Instead, some representatives of infraclass Chondrostei and Teleostei have flagella membrane fins (projections of the flagella membrane), which are also associated with doublets 3 and 8 of microtubules [19,47]. It is also worth mentioning that the flagella morphology of representatives of class Actinopterygii is extremely diverse [18].

Some parts of the fibrous sheath may be reduced in different groups of Chordata. For example, one of the longitudinal columns may be lost or reduced. Such an organization is observed in some representatives of the order Chimera (Figure 2(b11)) [16].

An interesting example of reorganization of the fibrous sheath may be found in group Reptilia. Thus, for example, in representatives of the order Squamata (Figure 2(b17)), the fibrous sheath includes the analogs of longitudinal columns—peripheral fibrils [48].

In the order Crocodiles, the nine dense fibrous chords are associated with triplets of microtubules of the distal centriole. In the principal piece of spermatozoa, only two of these structures are present and are associated with doublets 3 and 8 of microtubules (Figure 2(b24)) [29]. It is also worth mentioning that the area where fibrous cords are observed is extremely short [29].

The other representatives of the Reptilia have undergone a reduction in fibrous sheath structures. For example, in the order of Testudines, longitudinal columns are absent (Figure 2(b22)) [27]. The reduction in fibrous sheath structures continued in the descendants of Reptilia—birds. Representatives of the subclass Neognathae (Figure 2(b19)) lack both ribs and longitudinal columns [24].

There are also known examples of a complete reduction in fibrous sheath among groups of Amphibia. What is more interesting in these cases is that the function of the absent longitudinal columns is performed by the evolutionary unique structures called axial and juxta-axonemal fibers (Figure 2(b20)) [23]. They are considered by some authors a homologous structure to mammalian LC [23] and to fibrous cords of crocodiles [29]. The spermatozoa of all these animals are supplied with an undulating membrane. It reaches 3 μm in width and has a central lamina connecting to the axial fibril. The axial fiber plays the role of stiffener of the undulating membrane, while juxta-axonemal fibers are used by some species of amphibian to anchor the structure to the axoneme.

Among amphibians, different variants of these structures may be observed. If the undulating membrane is anchored by a juxta-axonemal fiber (like in order Anura), then it can be associated with the three doublets of microtubules or several at once. For example, in Discoglossus pictus, it is anchored to two, three, and four doublets [49]. In the order Urodela, in contrast to Anura, the axial and juxta-axonemal fibers are located on opposite sides of the axoneme: the axial fiber is localized near two or three doublets, and the juxta-axonemal fiber is associated with eight doublets [34] (Figure 2(b29)). The representatives of the Gymnophiona possess only the axial fiber, which is associated with three doublets of microtubules (Figure 2(b18)) [23]. 

In mammalian spermatozoa, in addition to the fibrous sheath, nine intermediate filament-like structures called outer dense fibers (ODFs) are located in flagella. Each of these fibers is paired with the microtubule doublet of the axoneme [50]. The length of the ODFs varies. Those associated with doublets 1, 5, and 6 are the longest, spanning about three-quarters of the flagellar length, while those associated with doublets 3 and 8 are the shortest, ending at the transition of the midpiece to the principal piece [51], where they are replaced by longitudinal columns of the fibrous sheath [52]. The end piece lacks ODF [52].

The ODFs are anchored in a structure called the connecting piece, located at the base of the flagellum in mammals [53]. During spermiogenesis, the basal body of the flagellum is lost, so outer doublets lack structural support [53]. Consequently, when these doublets slide or are under tension, the ODF is used to stabilize them and redistribute the forces acting on them to the connecting piece [52].

The ODFs and fibrous sheath of the mammalian sperm significantly enhance the stiffness of the sperm tail in comparison to a relatively simple flagellum, such as that observed for sea urchin sperm. This results in the flagellum being harder to bend, necessitating the involvement of a greater number of dyneins. This, in turn, leads to the creation of longer bends and a greater wavelength during flagellar beating [52]. This hypothesis is supported by the observation that the dyneins of a bull or rat sperm have the same spacing on the doublets as in a sea urchin sperm and other invertebrate cilia and flagella. However, the wavelength during flagellar beating differs [52,54].

Furthermore, ODF enables an enhancement in the bending torque of the flagellum, achieved through a rise in the effective diameter in the proximity of the base of the flagellum. In certain mammalian species, including rats and hamsters, the distance between the ODFs in the midpiece is three to four times greater than that between the doublets (Figure 2c) [36]. At the same time, in marsupials, the spacing between the ODFs in the midpiece is six times the diameter of the central axoneme (Figure 2c) [35]. In each case, the significant expansion of the effective diameter of the flagellum amplifies the bending torque by a similar proportion [52].

In mammalian sperm, there is an increase in the number of dyneins participating in bend formation and the effective diameter for torque development. These are undoubtedly adaptations facilitating the bending of the large and stiff flagellum, which is a characteristic feature of mammalian species [52].

Thus, the structure of spermatozoa, particularly its tail, varies dramatically among different taxonomic groups of Metazoa. Nevertheless, we still can trace some common patterns; for example, stiffeners of fibrous sheath are associated with doublets 3 and 8 of microtubules in the majority of species. Thereby, there may be some evolutionary-conserved mechanisms to mark these doublets and position stiffeners.

## 3. Proteins of Fibrous Sheath and Associated Structures

The fibrous sheath consists of electron-dense material, and among its main functions are the limitation of the flagellum bending planes and the anchoring of signaling and glycolytic proteins. Different protein groups, which are associated with fibrous sheath, will be discussed below (Table 1).

### 3.1. Protein Kinase-Associated Proteins

cAMP is a second messenger that plays an important role in mature spermatozoa; for example, it enhances its motility [55] and induces the acrosome reaction [56]. In mammalian cells, one of the most essential targets of cAMP is cAMP-dependent protein kinase A (PKA) [57]. At the same time, in the spermatozoa of sea urchins and fish [58], both the PKA and PKC enzymes play a role in regulating sperm motility through a complex interplay within the signaling pathway [59]. The inactive PKA is a tetrameric serine/threonine kinase that consists of two regulatory (R) subunits and two catalytic (C) subunits. When cAMP binds to PKA, it breaks [60] the circuitry of cooperative interactions stemming from the cAMP-binding pocket, thereby uncoupling subdomains of R subunits and causing disruption of protein–protein binding between R and C subunits, leading to activation of PKA through dissociation of R subunits [60].

There are several isoforms of PKA and a family of different R subunits (RIα, RIβ, RIIα, RIIβ) and C subunits (Ca, Cb, Cg, PRKX). Two types of R subunit are expressed in testis: Riα; and RIIα. Although RIα can be detected throughout all spermatogenesis, RIIα, which can be first detected in elongating spermatids, is the predominant type of R subunit in mature spermatozoa [61]. Disruption of R subunits leads to uncontrollable cell division; as an example, loss of function mutation in RIα results in Carney syndrome [62].

Ca subunit has two isoforms (Ca1 and Ca2), which are both encoded by the PRKACa gene [63]. The full-length transcript leads to Ca1 synthesis, but there is an alternative promoter that activates after the pachytene stage, resulting in a synthesis of a shortened Ca2, lacking the first few amino acids [64]. Although the predominant C subunit in germ cells is Ca1, Ca2 takes its place after the pachytene stage [64]. While Ca1 is ubiquitously expressed, Ca2 is specifically expressed in mature sperm and spermatocytes [65]. Knockout mice for PRKACa exhibit a slower growth rate, and most of them (~75%) do not survive the early postnatal period [66]. Mice lacking only Ca2 isoform show a normal phenotype, except being completely infertile as a result of decreased motility of spermatozoa [63].

**Figure 3:**
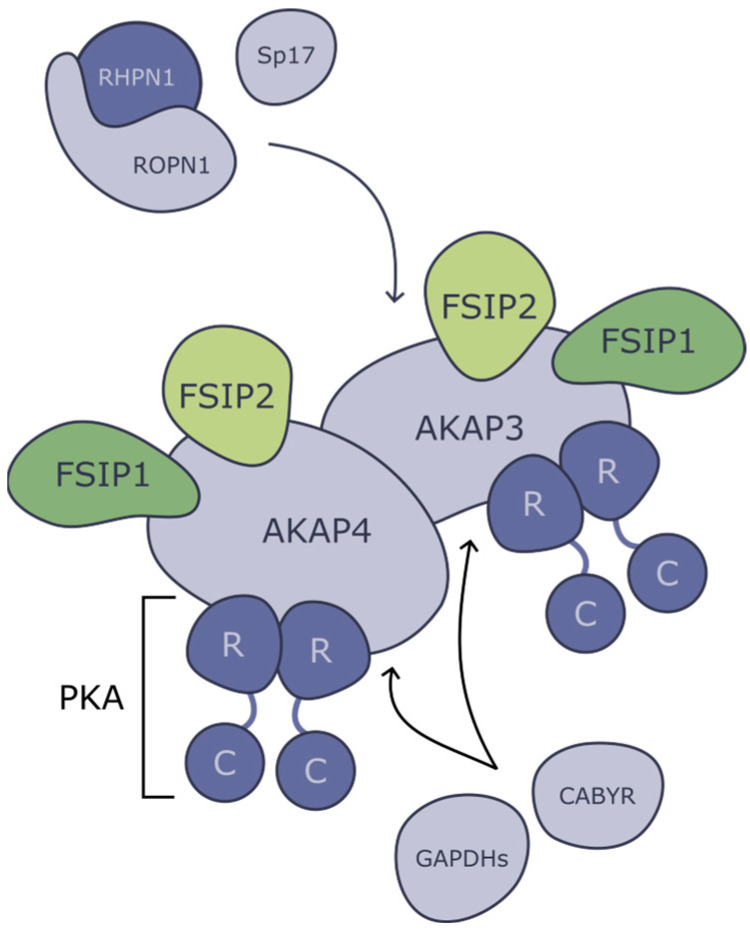
Interactions between main proteins in fibrous sheath. AKAP3 and AKAP4 can both bind PKA via its R subunits. AKAP4 can also bind AKAP3, and both of them can interact with FSIP1 and FSIP2. ROPN1 and Sp17 can interact only with AKAP3, and RHPN1 can interact with ROPN1. CABYR and GAPDHs are thought to interact with both AKAPs.

There is a mechanism to enhance the activity and specificity of PKA by anchoring it together with its target. This process is enabled by scaffold proteins, such as A-kinase-anchoring proteins (AKAPs) in mammals (Figure 3). They bind PKA via its R subunits and anchor it in the cytoskeleton or to subcellular organelles in proximity to PKAs’ target proteins. There are more than 50 known members of the AKAP family (including splice variants). Some of them, such as AKAP450, are expressed throughout the organism, while others demonstrate tissue or cell-type specificity [67]. In addition, in fish, a similar mechanism of PKC anchoring that does not involve the proteins of the fibrous sheath has been identified. PKC is directly attached to the flagellar axoneme via the outer dynein arm of the flagellar axoneme, where it regulates sperm motility by phosphorylating a dynein subunit [68].

In mammals, the sperm fibrous sheath (FS) is mainly composed of members of the AKAP protein family. However, in some animals lacking FS, AKAPs can still be observed in the spermatozoa. In bony fish, a protein similar to human AKAP7 has been shown to be phosphorylated during the initiation of sperm motility [69], but a detailed explanation of its function in this process remains to be found.

**Figure 4:**
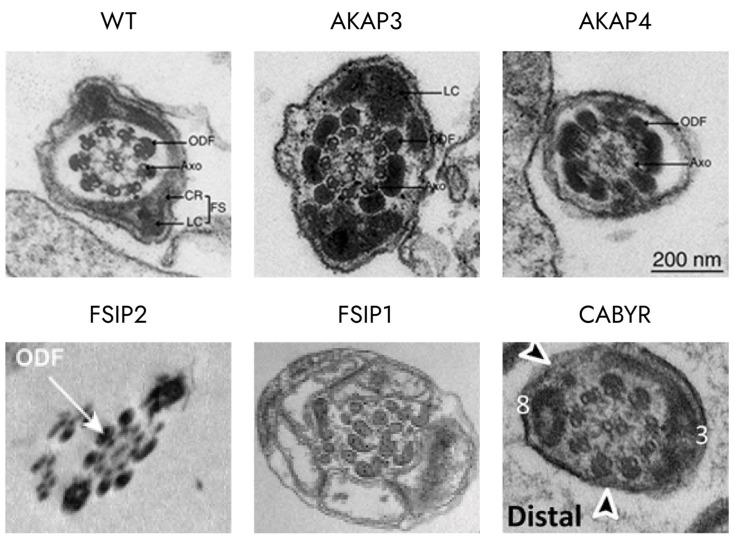
Abnormalities in the morphology of the murine sperm flagellum caused by defects in the structural proteins of the fibrous sheath. Normal structure of sperm tail is demonstrated in WT [70]. Deficiency in AKAP3 [70] results in loss of circular ribs (CR) in between longitudinal ribs (LC), while deficiency in AKAP4 [70] leads to complete loss of FS with only some electron-dense material left adjacent to the plasma membrane. In FSIP2 [73], deficiency fibrous sheaths were absent, and the axonemes were exposed. FSIP1 [77] knockouts demonstrated misassembly of the main ultrastructural components of sperm flagella, such as mitochondria, outer dense fiber, and “9 + 2” axonemal structures. In CABYR [84] knockouts, transverse ribs of the fibrous sheath were preferentially disrupted or expanded. Numbers indicate the doublets of axoneme. Black arrows indicate the disrupted FS.

In mice, AKAP3 is important for the formation of specific subcellular structures, primarily the circumferential ribs (CRs) of the FS [70], whereas AKAP4 affects the formation of the entire FS [4]. It has been shown in a yeast model that both of them are able to bind R subunits of PKA (AKAP4 even has two binding sites for PKA [71]), and additionally, AKAP4 can bind AKAP3 [72]. The knockout mice for both genes are sterile, and their abnormal spermatozoa are characterized by disrupted FS and shortened principal pieces of the sperm tail [70] (Figure 4).

From knockout phenotypes, it appears that the main role of AKAPs is the structural integrity of flagella; however, they are also involved in a great variety of signaling pathways that take place in sperm tails. AKAPs interact with proteins that share homology with PKA R subunits; thus, AKAPs serve not only as a scaffold for PKA and its substrate but also as big and branched machinery of different proteins. For example, AKAP4 and AKAP3 bind two fibrous sheaths interacting proteins (FSIP1 and FSIP2) [72].

FSIP2 is one of the largest proteins (6907 amino acids) in the human genome and is also one of the major components of the fibrous sheath [73]. Mutations in FSIP2 lead to impaired formation of flagella, which is characterized by a completely disorganized FS, with abnormalities in microtubules and dynein arm-associated proteins [74,75].

It is not fully clear how FSIP2 interacts with AKAPs (Figure 3); however, it is essential for normal FS development and functioning. It was proposed that FSIP2 interacts with AKAP4, and this scaffold then attracts additional proteins such as ROPN1, CABYR, and AKAP3 [73]. FSIP2 has two binding sites for AKAP4, and AKAP4 must bind them simultaneously to form the normal FS and sperm flagella [73]. If FSIP2 is truncated and the second binding site is lost, the interaction with AKAP4 will be significantly impaired, leading to male infertility [73].

Moreover, it was shown that FSIP2 deficiency induces the elongation of the mitochondrial sheath [76]. It was suggested that FSIP2 may be involved in the process of mitochondria division during meiosis and early spermatogenesis or the process of the retention of mitochondria during the differentiation of the round spermatids into elongated spermatids [76]. 

Also, in individuals with FSIP2 deficiency, the annulus ring was eliminated, and IFT-B-related proteins (IFT88, IFT74, and IFT20) were dislocated or absent, which can be an indication of FSIP2’s role in intraflagellar transport [76].

At the same time, another member of the FSIP family, FSIP1, is shown to interact with intraflagellar transport machinery; in particular, it can interact with IFT20 [77]. *Fsip1^−/−^* mice were infertile, with a low sperm count and impaired motility [77]. Electronic microphotographs of flagella ultrastructure of *Fsip1^−/−^* revealed disassembly of mitochondria, outer dense fiber, and axonemal structure [77] (Figure 4).

Ropporin (from Japanese “oppo”—tail) is a testis-specific protein that is located in FS, mainly on its inner surface [78]. Ropporin is expressed exclusively in the testis and, specifically, in germ cells at the late stage of spermatogenesis. Ropporin has an N-terminal sequence that shares homology with the RIIα subunit of PKA and allows it to bind AKAP3 [78] (Figure 3). At the C-terminus, Ropporin has a PDZ domain involved in binding rhophilin (ROPN1), another testis-specific protein, that serves as a putative target of small GTPase Rho [78]. It is also expressed in the germ cells at the late stage of spermatogenesis and localized in sperm flagella [78]. Moreover, it was demonstrated that rhophilin tends to localize mostly on the outer surface of the outer dense fiber and less on the surfaces of the fibrous sheath [78]. Therefore, it is likely that rhophilin and ropporin may form a temporal bridge between the outer dense fiber and the fibrous sheath. Also, it was supposed that most of the ropporin is buried deep into FS and is not accessible for antibodies. 

It is worth mentioning that in the described example, AKAP3 serves as a scaffold between PKA signaling and Rho signaling. GTPase Rho regulates a number of cell processes, including hyperactivation and sperm motility [78]. 

There is another protein that shares much in common with rhophilin—rhophilin-like protein (ROPNL1). It was shown that *Ropn1^−/−^* mice are subfertile, while mice with double knockout of both Ropn1 and Ropnl1 are infertile [79] (Figure 4).

Besides rhophilin, ropporin can bind Ca^2+^-binding tyrosine-phosphorylation-regulated protein (CABYR) [80,81]. As implied by its name, CABYR is a calcium-binding protein that loses its binding ability after phosphorylation. CABYR is phosphorylated when sperm undergoes capacitation, which results in an increase in calcium concentrations, contributing to the hyperactivation of spermatozoa [82]. Moreover, CABYR plays an important role in FS development. It also has an N-terminal domain that shares high homology with the RIIα domain of protein kinase A (PKA), which allows it to bind to AKAPs [83]. In *Cabyr^−/−^* mice, the fibrous sheath is disorganized; circumferential ribs are disrupted, and cytosolic space between the axoneme and the plasma membrane is increased [84] (Figure 4).

Calcium signaling is a very important part of capacitation. Besides CABYR, there is a handful of sperm-specific Ca^2+^-binding proteins, which are essential to flagellar beating. As an example, Ca^2+^ ions regulate calmodulin-dependent and cAMP-dependent protein phosphorylation that activates the axoneme [85]. Some findings show that binding of Ca^2+^ directly to a component of the dynein complex regulates ATP-sensitive interactions between the dynein heavy chain and microtubules, which is, indeed, important for proper motility [86]. It is also interesting to notice that microtubules themselves can serve as transmitting lines for pulses of calcium ions [86].

Sperm protein 17 (Sp17) is another example of an FS protein that can bind to the members of the AKAP protein family. Although Sp17 was originally thought to be gamete-specific, mRNA encoding Sp17 has been found in a range of murine and human somatic tissues [87]. Because of Sp17’s ability to bind sulfated carbohydrates, Sp17 was thought to be responsible for interaction with Zona pellucida [88], but later, it was discovered that Sp17 is localized in the sperm tail and should have additional functions [87].

Sp17 has three domains: (1) a highly conserved N-terminal domain that shares 45% similarity with the human RIIα of PKA; (2) a central sulfated carbohydrate-binding domain; (3) a C-terminal Ca^2+^/calmodulin (CaM) binding domain. The first domain gives Sp17 the ability to bind AKAPs, which may indicate its structural function in the FS. Also, Sp17 has been shown to function immediately after the beginning of the acrosome reaction as a zona pellucida binding protein and to lose its C-terminal Ca^2+^/calmodulin (CaM) binding domain, which may be attributed to calcium signaling incapacitation, as CABYR [89].

The filament-like protein (FS39) is also a part of the structure of the sperm tail and a component of its fibrous sheath. It colocalizes with the AKAP82 and is thought to be one of the targets of phosphorylation events that are important for sperm motility [90]. However, the exact role of FS39 and the effect of chemical modifications, particularly phosphorylation, on its function remains unknown [90].

### 3.2. Glycolytic Enzymes

The movement of the flagella requires a lot of energy in the form of ATP. Although oxidative phosphorylation is more efficient than glycolysis, mitochondria from the middle piece may not be able to provide ATP for all the length of the sperm tail, as diffusion speed is limited. In addition, it was shown that mice lacking the testis-specific cytochrome cT, the only cytochrome c in sperm mitochondria, are fertile [91]. This phenomenon underlines the importance of glycolysis in sperm energy supply. Indeed, glycolysis is essential for sperm motility, and there are sperm-specific isozymes of glycolytic enzymes [92]. For example, glyceraldehyde 3-phosphate dehydrogenase-S (GAPDS) is an enzyme that converts glyceraldehyde-3-phosphate to 1,3-bisphosphoglycerate. The enzymatic steps prior to GAPDH action utilize two molecules of ATP, and only reactions after GAPDH return four molecules of ATP, making all glycolysis profitable. Inhibition of GAPDH will not only disrupt glycolysis but also turn it into an energy-consuming process. GAPDH is localized on the ribs of the FS of the principle piece and is expressed and incorporated in the FS at the late steps of spermatogenesis [93,94].

The ultrastructure of spermatozoa flagella of *Gapds^−/−^* mice is normal; however, sperm overall is completely immotile, resulting in mice infertility [95]. Moreover, for *Gapds^−/−^* mice, it was shown that even in the presence of both pyruvate and lactate, they remained motionless [95]. This fact supports the idea that the predominant energy supply system of spermatozoa is glycolysis.

Some sperm-specific glycolytic enzymes are tightly bound to FS (GAPDS [96], lactate dehydrogenase A, aldolase A [97], and pyruvate kinase [98]). It was also shown that hexokinase1-s (HK1-s) and phosphofructokinase (PFK) are bound to FS by glutathione S-transferase mu class 5 (GSTm5) [99]. This compartmentalization of glycolytic enzymes in FS may positively influence the overall effectiveness of glycolysis, which is essential for flagella beating.

### 3.3. Intraflagellar Transport

Assembly of such a complex structure as flagella depends on a transport system known as ‘intraflagellar transport’ (IFT), which implements the transport of non-membrane-bound particles between the cell body and the tip of the cilium or flagellum.

The main participants of IFT are the IFTa and IFTb protein complexes, which together form a “train” that carries diverse cargoes to the cilia. The assembly of IFT components starts with the polymerization of IFTb complexes at the base of the cilia and proceeds with the polymerization of IFTa on this platform. As soon as this core structure is assembled, it recruits kinesin-2 motors and some other signaling cargoes, as well as autoinhibited cytoplasmic dynein-2 motors. Using kinesin as the main motor, the IFT train moves to the end of the cilia, where its cargoes dissociate into the cilia. The IFTa/b components then remodel into a conformationally distinct retrograde train, which rebinds to the now-active dynein-2 and transports a new selection of cargoes back to the cell body [100].

Nowadays, 20 proteins of IFT are studied, and it was shown that disruptions in their functioning may lead to defects in spermatozoa development; several of them will be discussed below.

IIFT74-IFT81 dimer is a core component of the IFTb complex, which is responsible for the transport of β-tubulin to the place of the microtubule assembly during the formation of the axoneme in differentiating spermatids [101]. Male mice without IFT74 are sterile, with a variety of abnormalities in the sperm, including very short tails and abnormally shaped heads. Mutations in IFT74 are associated with diverse ciliopathies in humans (for example, Joubert syndrome [102]), and they can also induce male sterility due to impaired flagellogenesis [103]. Mutations in IFT81 are also associated with ciliopathies [104,105].

RABL2 is a small GTPase that is essential for normal ciliogenesis. In its GTP-bound state, RABL2 can interact with the IFTb complex via the IFT74-IFT81 dimer, which leads to hyperactivation of RABL2 [106] and promotes the initiation of anterograde IFT [107]. Although *Rabl2^−/−^* mice are infertile and demonstrate decreased sperm motility, no structural abnormalities in spermatozoa morphology have been detected.

IFT88 is another IFTb protein that is essential for proper axoneme formation. *Ift8^−/−^* mice are sterile, and their spermatids exhibit severe developmental defects, for example, truncated axoneme, lack of fibrous sheath longitudinal columns, accumulation of fibrous sheath rib material, and ectopically assembled outer dense fibers and microtubules [28].

### 3.4. Post-Translational Modifications

Chemical modifications such as glycosylation, acetylation, and attachment of amino acid residues have an important effect on the protein molecules that make up flagellar microtubules. Post-translational modifications of tubulin affect microtubule structure and sperm motility [92].

Tubulin tyrosine ligase-like (TTLL) is a family of proteins involved in such chemical modifications of spermatozoa tubulin as glycylation (TTLL3, TTLL8) and polyglutamylation (TTLL1, TTLL9, TTLL4, TTLL5) [108].

**Figure 5:**
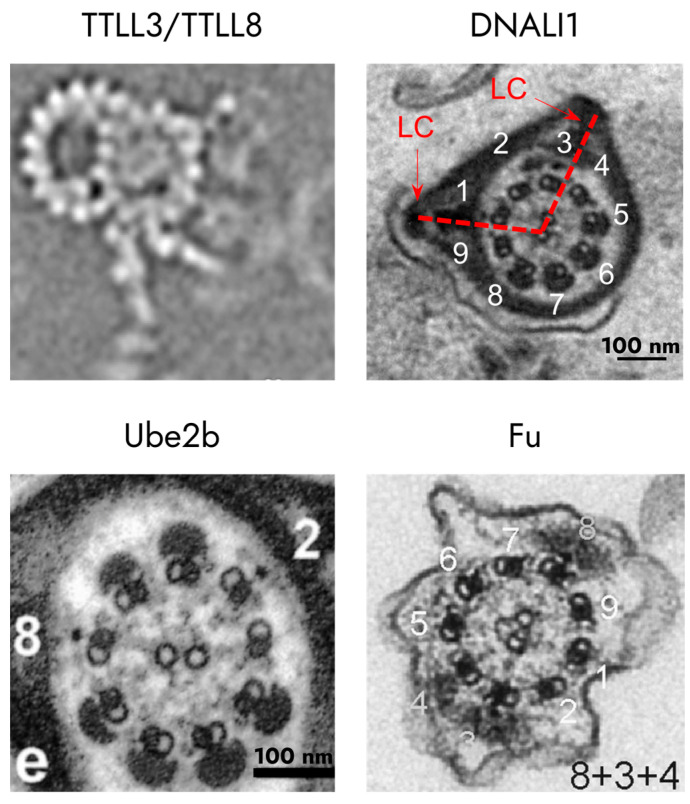
Phenotypes of mouse spermatozoa caused by defects in post-translational modifications of structural proteins of the fibrous sheath. *Ttll3^−/−^ Ttll8^−/−^* mouse models lacking tubulin glycylation did not show any evident changes in axoneme assembly at the macromolecular level. [110] The flagella of male *Dnali1^−/−^* mice exhibited asymmetry in the position of longitudinal columns (shown by red lines). [111] In *Ube2b^−/−^* mice, there is a shift in the position of the longitudinal columns; thus, in the figure, one of the columns is bound to doublet 2 instead of doublet 3 [112]). Spermatozoa of the Vasa-Fu genotype showed an abnormal number of longitudinal columns, such as at positions 8+3+4, where position 4 is redundant [113]. Numbers indicate the doublets of axoneme. Red arrows indicate the position of LC.

Glycylation involves the modification of dynein molecules with glycine residues and is carried out by the enzymes TTLL3 and TTLL8 [109]. Mice lacking functional copies of both of these genes (*Ttll3^−/−^Ttll8^−/−^*) exhibit spermatozoa with defects in flagella beating, which results in an overall reduction in sperm motility and abnormal circular trajectory that significantly differs from the wild type. But, on the ultrastructural level, the described knockout sperm exhibits disturbances in the axonemal dynein arms formation (Figure 5) [110].

Other representatives of the TTLL family (TTLL1, TTLL9, TTLL4, TTLL5) catalyze tubulin polyglutamylation in flagella [114]. The Ttll1 knockout (*Ttll1^−/−^*) in mice leads to the reduction in the polyglutamylation of flagella proteins, which results in defective spermatozoa with shortened tails [115]. It was shown that α- and β-tubulins of the *Ttll1^−/−^* mice axoneme lacked most of the polyglutamylation, while the level of other modifications was not significantly altered [116].

*Ttll9^−/−^* mice are also infertile and exhibit defects in sperm motility. While TTLL1 is involved in the polyglutamylation of axonemes overall, TTLL9 predominantly influences the polyglutamylation of the fifth and distal regions of the seventh doublets of microtubules [117]. It was proposed that TTLL9 participates in the formation of polyglutamylation heterogeneity and, thereby, maintains the normal structure and curvature of flagella [117].

TTLL4 and TTLL5 catalyze the synthesis of γ-carboxyl linkages for β- and α-tubulins in mice and humans [118]. Although TTLL4 knockout mice are fertile and have normal sperm shape [119], TTLL5 knockout in mice leads to a significant decrease in the amount of polyglutamylated tubulin and a complete reduction in four doublets of axonemal microtubules in 95% of cases [120].

Thus, TTLL family enzymes are essential for sperm flagella assembly; however, the detailed mechanisms of their action have to be studied.

The importance of maintaining a certain degree of polyglutamylation for spermatogenesis is proved by the fact that excessive glutamylation leads to spermatogenesis disorders. The tubulin deglutaminase CCP5 (also known as AGBL5) plays a crucial role in the regulation of polyglutaminylation during spermatogenesis in mice [121]. CCP5 knockout in mice has been shown to result in the accumulation of polyglutaminated tubulin, which is accompanied by the appearance of disorganized microtubules in the flagella [121] and abnormal manchette formation [121]. In another study, *Ccp5^−/−^* spermatozoa exhibited an abnormally shaped head as well as incompletely sheathed flagella [122].

Acetylation is another important enzymatic modification of sperm proteins. HDAC6 and CDYL proteins are involved in this process. HDAC6 (histone deacetylase-6) is a tubulin deacetylase located predominantly in the caudal part of the flagella [123]. Loss of function mutations in *Hdac6* leads to the hyperacetylation of α-tubulin, but otherwise, it does not cause any significant phenotypic changes in mice [124]. Inhibition of HDAC6 (Trichostatin A, Tubastatin A, and sodium butyrate) has been shown to increase α-tubulin expression and influence the stability of microtubules in flagella, affecting sperm motility [125].

As for CDYL, it predominantly colocalizes with acetylated alpha-tubulin in sperm flagella. Mice lacking CDYL produce spermatozoa with deformed heads and exhibit significant germ cell death, which alters spermatogonia, spermatocytes, and spermatid numbers [126]. 

Phosphorylation is one of the most significant post-translational modifications to which numerous sperm proteins are susceptible [127]. As previously outlined, one of the pivotal proteins responsible for this modification within the fibrous sheath is PKA, which acts as an effector in numerous signaling pathways [57]. Its activity is predominantly associated with capacitation in mature sperm. During capacitation, a considerable number of sperm proteins undergo tyrosine phosphorylation, for instance, AKAP3 (mouse) [45], AKAP4 (mouse) [45], CABYR (mouse) [45], dynein light (*Chum salmon* [128], sea urchin [129], Ascidia [130]), and heavy (mouse [131] and sea urchin [129]) chains. It is worth noticing that phosphorylation patterns are species-specific. For instance, AKAP4 is phosphorylated at ser/thr residues in mouse sperm rather than at tyrosine residues, as observed in humans [132] and hamsters [133]. 

Moreover, phosphorylation also plays an important role during tail formation. Recently, serine/threonine kinase 33 (STK33) was found to phosphorylate AKAP3 and AKAP4, and loss-of-function mutation in this gene leads to oligoasthenozoospermia in humans and mice [134]. At the same time, knockouts display a broad phenotype, which includes structural abnormalities of flagella, including misarrangement of mitochondria and fibrous sheath, partial loss of ODF, and a lack of microtubules in the axoneme [134].

### 3.5. Protectors from Oxidative Stress

Maintenance of proper redox potential is crucial for normal spermatogenesis. Small amounts of reactive oxygen species (ROS) are thought to play an important role in sperm maturation and capacitation [135]; however, high levels of ROS can disrupt this process, leading to infertility [136]. The epididymis and seminal vesicles are considered the main antioxidant-secretion organs [137], which additionally points to the importance of ROS control in spermatogenesis. 

Spermatozoa and seminal plasma contain a lot of proteins and small molecules that lower ROS levels. Besides well-known enzymes, such as superoxide dismutase, catalase, and glutathione reductase [138], there are some testis-specific proteins that additionally protect male germ cells from oxidative stress.

SPTRX-1 (Sperm-specific Thioredoxin-1, also called TXNDC2) is a member of the thioredoxin protein family. In general, thioredoxins are small redox proteins that are known to reduce disulfide bonds, thereby serving as a protective agent against oxidative stress. Thioredoxin proteins have been associated with sperm maturation and are thought to assist in the correct formation of disulfide bridges in protamines, which replace histones during the hypercondensation of sperm DNA [139].

SPTRX-1, as sperm-specific thioredoxin, likely contributes to the redox regulation critical for maintaining sperm function and integrity. Through its potential to mitigate oxidative damage and regulate disulfide bond formation, it may help to keep spermatozoa viable and functional, thus ensuring the male reproductive capacity [140]. The expression pattern of SPTRX-1 during rat spermatogenesis suggests that it could be a part of a nucleation center for the formation of the longitudinal columns and circumferential ribs [141].

Glutathione-S-transferases (GST) also play an important role in protecting cells from oxygen stress [142]. GSTs are involved in a number of processes, but their main purpose is detoxification. GSTs are eliminating xenobiotics using a reduced form of glutathione (GSH), catalyzing nucleophilic attack by GSH on xenobiotics. As discussed previously, there is a testis-specific μ-class GSTm5, which is located in FS and participates in the compartmentalization of HK1s [99]. GSTm5 is first expressed in the meiotic phases of male germ cell development, and its expression is restricted to spermatogenic cells [143].

### 3.6. Proteins Affecting the Positioning of Longitudinal Columns

As was mentioned above, the structure of flagella of various representatives of Metazoa can be extremely diverse. The method of attachment of the flagellum to the middle part, the structure of the axoneme, as well as the organization of surrounding structures may vary. In this review, we focus more on the structure of the fibrous sheath.

This structure first appears in vertebrates and acquires different features in different groups of animals. So, for example, one can trace the gradual reduction in the components of the fibrous sheath in reptiles and their descendants—birds. Crocodiles and lizards [19,47], as well as ancient birds like emu and tinamou, have all the main components (ribs and longitudinal columns) of fibrous sheath [52]. However, in all representatives of the Neognathae, the structure of the fibrous sheath is simplified, and ribs and longitudinal columns are absent. [24]. As was described above, in many groups of animals, the fibrous sheath includes structures that perform the function of stiffeners, and it is especially interesting that in the vast majority of cases, they are associated with axoneme doublets 3 and 8. However, the molecular mechanism of the positioning of these structures still remains unknown.

Participants of the positioning path of the longitudinal columns to microtubule doublets 3 and 8 may be discovered in the future among the proteins that appeared in vertebrates at the same time with the development of fibrous sheath (Figure 5). For example, for the structural proteins AKAP3 and AKAP4, which arise in vertebrates along with the fibrous sheath, an association with the formation of components of the fibrous sheath was confirmed. The knockout of the Akap4 gene leads to the complete disappearance of the longitudinal columns of the fibrous sheath in mouse sperm [4], while, at the same time, an impaired formation of circumferential ribs is observed in *Akap3^−/−^* mice [144].

Transport and modifying proteins are of particular interest in the context of studying the positioning of the longitudinal columns and circumferential ribs of the fibrous sheath. For example, it was shown that the absence of polyglutamination carried out by TTLL9 leads to the disappearance of the seventh doublet of microtubules in the axoneme [117], while the attachment of longitudinal columns is disrupted in the absence of ubiquitin ligase UBE2B. It is interesting that the columns themselves are formed normally; however, instead of joining to doublets 3 and 8, they are attached randomly and can even increase in number (Figure 5) [112].

A similar phenotype also was observed in knockout mice *Dnali1^−/−^* (Figure 5) [111,145]. This gene is present in all Metazoa, but in vertebrates, it has been shown to be involved in dynein-associated transport and the assembly of AKAP3 and AKAP4 [145]. The absence of this protein also leads to abnormalities in the attachment of the longitudinal columns of the fibrous sheath. Interestingly, a similar phenotype can also be found for knockouts of genes associated with kinesin transport. In Fu knockout mice, sperm have a normal axoneme, but the periaxonemal structures are disorganized (Figure 5) [113]. In particular, additional longitudinal columns appear in mutant spermatozoa. Accordingly, sperm immobility is associated precisely with disturbances in the development of periaxonemal structures. The observed phenotype could be explained, for example, by the connection of Fu with transport systems. Fu has been shown in vitro to interact with a component of the intramanchette transport system (Kif27) [113]. The general localization of these proteins in the perinuclear space suggests that their interaction has a regulatory function. It is likely that the Fu/Kif27 complex controls the transport of specific cargo (possibly within the manchette), such as additional components of the sperm flagellum [113].

## 4. Evolutionary Distribution of the Fibrous Sheath Associated Proteins among Metazoa

**Figure 6:**
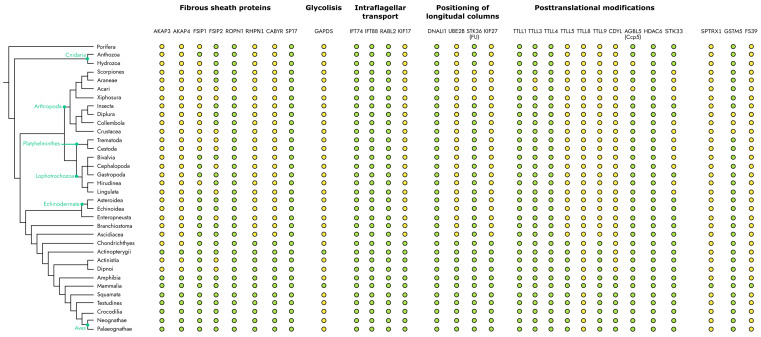
Evolutionary distribution of FS-associated proteins. Green circles indicate taxons in which the particular gene is present, yellow—in which the gene is absent. The phylogenetic tree was adopted from Schuster. H.C. [6]. The evolutionary distribution of specific genes was specified according to database OrthDB v11 [146].

During the consideration of the evolutionary representation of fibrous sheath proteins, one finds that although the structure itself originates in Vertebrates, many proteins are widely represented in diverse groups of Metazoa (Figure 6).

Three groups can be distinguished among the entire set of fibrous sheaths’ associated proteins: proteins that can be found in almost all Metazoa; the ones that are present only in Vertebrates; and proteins that are found exclusively in Mammals. It is interesting that the components of one functional system could have developed in different taxons.

The first group of proteins, which are widely represented in all vertebrates, includes some microtubule-modifying proteins (TTLL1, 4, 3, HDAC6, AGBL5), as well as proteins involved in the protein kinase signal transduction cascade (FSIP1, FSIP2, ROPN1, PRKACA).

Of particular interest is a group of proteins whose appearance coincides with the appearance of the fibrous sheath in animals. In particular, this group includes structural proteins of the fibrous sheath (AKAP3 and AKAP4), as well as some proteins that carry out post-translational modifications (TTLL5, TTLL9, UBE2B, CDYL) and components of transport systems (KIF17, KIF27).

Also, some proteins could be found exclusively in mammals, such as proteins associated with the protection of sperm from oxidative stress (GSTM5, TXNDC2) and that carry out specific glycilation of tubulin (TTLL8).

## 5. Conclusions

The ultrastructures of Metazoan spermatozoa are extremely diverse; however, as we have seen, there are homologous structures in various taxa. In this review, we focused on structures that attach to microtubule doublets 3 and 8, such as the longitudinal columns of mammalian FS [23], axial and juxta-axial fibers of amphibian [29], and flagella membrane fins of Teleostei [19,47]. This positioning is explained primarily by their mechanical functions to limit the movement of the flagellum in the plane perpendicular to the central pair of microtubules [3]. Since the first structures associated with these doublets appear in cartilaginous fish [15], it can be assumed that the molecular mechanism could have arisen approximately at the same time as the formation of vertebrates. This corresponds to the appearance of the FS in animals.

When considering the evolutionary distribution of proteins associated with FS, it can be noted that most of the genes, the mutations of which lead to incorrect positioning of the longitudinal columns of the FS [4,112,113,117], arose simultaneously with the appearance of the fibrous sheath. The described proteins are involved in IFT and related processes, have structural functions, and are involved in post-translational modifications in the flagellum.

Thus, positioning of the structural elements in sperm flagella is a complex process. It requires the proper functioning of various flagellum building systems. Moreover, this mechanism may include both components present in all metazoans and those that arose simultaneously with the appearance of the fibrous sheath. However, extensive research is needed to reveal the key components of LC positioning during FS assembly.

## Figures and Tables

**Table 1:** Proteins associated with fibrous sheath.

Protein	Cellular Localization	Function
Protein-kinase A *PKA*	Fibrous sheath	Phosphorylation of different targets, signaling
PKA-associated protein 3 *AKAP3*		Structural protein of circumferential ribs
PKA-associated protein 4 *AKAP4*		Structural protein of circumferential ribs and longitudinal columns
FS-interacting protein 1 *FSIP1*		Interaction with IFT machinery
FS-interacting protein 2 *FSIP2*		Structural protein of FS. Possibly involved in control of mitochondria
Ropporin		Binding of rhophilin
Rhophilin		Putative target of small GTPase Rho
Ca^2+^-binding Y-phosphorylation-regulated protein *CABYR*		Calcium signaling during capacitation, FS development
Sp17		Capacitation, structural protein of the FS
FS39		Structural protein of the FS
Glyceraldehyde 3-phosphate dehydrogenase-S *GAPDS*		Glycolytic enzyme
*IFT74*, *IFT81*	Intraflagellar transport	Core components of the IFT complex. Transport of β-tubulin
Rab-like 2 *RABL2*		Small GTPase. Initiation of anterograde IFT
*IFT88*		
Tubulin tyrosine ligase-like 3, 8 *TTLL3*, *TTLL8*	Axoneme	Tubulin glycylation
Tubulin tyrosine ligase-like 1, 9, 4, 5 *TTLL1*, *TTLL9*, *TTLL4*, *TTLL5*		Tubulin polyglutamylation
Cytoplasmic carboxypeptidase 5 *CCP5 (AGBL5)*		Tubulin deglutamination
Histone deacetylase-6 *HDAC6*		Tubulin deacetylation
Chromodomain Y Like *CDYL*		
Serine/Threonine kinase 33 *STK33*		Phosphorylation of AKAP3 and AKAP4
Sperm-specific Thioredoxin-1 *SPTRX-1*		Thioredoxin. Protection from oxidative stress, regulation of disulfide bond formation, FS formation
Glutathione S-transferase mu class *GSTm5*		Detoxification
Ubiquitin-conjugating enzyme E2 B *UBE2B*		Positioning of longitudinal columns
Dynein Axonemal Light Intermediate Chain 1 *Dnali1*		Dynein-associated transport and the assembly of AKAP3 and AKAP4
Fused *Fu*		Kinesin transport
